# Prevalence and Mortality due to *Mycobacterium tuberculosis* Bloodstream Infection in Adults With HIV: A Multicountry Prospective Cohort Study

**DOI:** 10.1093/ofid/ofag424

**Published:** 2026-07-07

**Authors:** Bianca Sossen, Madalo Mukoka, Rita Székely, Monde Muyoyeta, Elizabeth Nakabugo, Jerry Hella, Hung Van Nguyen, Sasiwimol Ubolyam, Marcia Vermeulen, Chad M Centner, Sarah Nyangu, Nsala Sanjase, Mohamed Sasamalo, Huong Thi Dinh, The Anh Ngo, Weerawat Manosuthi, Supunnee Jirajariyavej, Nhung Viet Nguyen, Anchalee Avihingsanon, Claudia M Denkinger, Klaus Reither, Lydia Nakiyingi, Andrew D Kerkhoff, Morten Ruhwald, Graeme Meintjes, Peter MacPherson

**Affiliations:** Department of Medicine, Faculty of Health Sciences, University of Cape Town, Cape Town, South Africa; Institute of Infectious Disease and Molecular Medicine, University of Cape Town, Cape Town, South Africa; Public Health Group, Malawi-Liverpool-Wellcome Programme, Blantyre, Malawi; Department of Pathology, Kamuzu University of Health Sciences, Blantyre, Malawi; FIND, Geneva, Switzerland; Laboratory for Intelligent Global Health and Humanitarian Response Technologies, EPFL, Lausanne, Switzerland; Centre for Infectious Disease Research in Zambia, Lusaka, Zambia; Infectious Diseases Institute, College of Health Sciences, Makerere University, Kampala, Uganda; Ifakara Health Institute, Dar es Salaam, Tanzania; National Lung Hospital, Ha Noi, Viet Nam; HIV-NAT, Thai Red Cross AIDS and Infectious Diseases Research Centre, Bangkok, Thailand; Center of Excellence in Tuberculosis, Faculty of Medicine, Chulalongkorn University, Bangkok, Thailand; Wellcome Center for Infectious Diseases Research in Africa, Institute of Infectious Disease and Molecular Medicine, University of Cape Town, Cape Town, South Africa; Division of Medical Microbiology, University of Cape Town, Cape Town, South Africa; National Health Laboratory Service, Groote Schuur Hospital, Cape Town, South Africa; Centre for Infectious Disease Research in Zambia, Lusaka, Zambia; Centre for Infectious Disease Research in Zambia, Lusaka, Zambia; Ifakara Health Institute, Dar es Salaam, Tanzania; National Lung Hospital, Ha Noi, Viet Nam; Viet Tiep Hospital, Hai Phong, Viet Nam; Bamrasnaradura Infectious Diseases Institute, Nonthaburi, Thailand; Taksin Hospital, Bangkok, Thailand; National Lung Hospital, Ha Noi, Viet Nam; HIV-NAT, Thai Red Cross AIDS and Infectious Diseases Research Centre, Bangkok, Thailand; Center of Excellence in Tuberculosis, Faculty of Medicine, Chulalongkorn University, Bangkok, Thailand; Division of Infectious Disease and Tropical Medicine, Heidelberg University, Medical Faculty, Heidelberg, Germany; Faculty of Medicine, Heidelberg University, Heidelberg, Germany; German Centre for Infection Research, Partner Site Heidelberg University Hospital, Heidelberg, Germany; Swiss Tropical and Public Health Institute, Allschwil, Switzerland; University of Basel, Basel, Switzerland; Infectious Diseases Institute, College of Health Sciences, Makerere University, Kampala, Uganda; Division of HIV, Infectious Diseases, and Global Medicine, Zuckerberg San Francisco General Hospital and Trauma Center, University of California San Francisco, San Francisco, California, USA; FIND, Geneva, Switzerland; Novo Nordisk Foundation Initiative for Vaccine and Immunity, NIVI-Development, Panum Institute, Copenhagen, Denmark; Department of Medicine, Faculty of Health Sciences, University of Cape Town, Cape Town, South Africa; Institute of Infectious Disease and Molecular Medicine, University of Cape Town, Cape Town, South Africa; Blizard Institute, Faculty of Medicine and Dentistry, Queen Mary University of London, London, UK; School of Health & Wellbeing, University of Glasgow, Glasgow, UK

**Keywords:** HIV, MTB bloodstream infection, multinational, survival analysis, tuberculosis

## Abstract

**Background:**

HIV-associated tuberculosis is a leading cause of mortality. *Mycobacterium tuberculosis* bloodstream infection (MTB-BSI) is complicated by diagnostic difficulty and severe illness. We assessed whether MTB-BSI continues to be common in the era of widespread antiretroviral therapy and whether it continues to result in increased risk of early mortality.

**Methods:**

We enrolled inpatients from medical wards irrespective of tuberculosis symptoms and outpatients with tuberculosis symptoms. All participants had HIV and were ≥18 years old, and all were recruited from 7 countries: Malawi, South Africa, Tanzania, Thailand, Uganda, Vietnam, and Zambia. Participants also had <3 doses of antituberculosis treatment in the past 60 days and no isoniazid preventive therapy in the past 6 months. We estimated the prevalence of MTB-BSI. In Bayesian survival models, we estimated the hazards of mortality for inpatients with MTB-BSI vs those without.

**Results:**

Between 2019 and 2021, 1703 participants were included: 44% were hospitalized, the median CD4 count was 361 cells/μL, and 77% reported using antiretroviral therapy. Crude prevalence of MTB-BSI varied by country but was 4.4% (32/723) among all inpatients, 22.5% (32/142) among inpatients with microbiologically confirmed TB, and 0.2% (2/913) among all outpatients. Among all inpatients, those with MTB-BSI had 2.65-times (95% credible interval, 1.06–5.01) increased hazard of death by 30 days and 1.79-times (95% credible interval, .81–3.11) increased hazard by 70 days. Lower CD4 and older age were strongly associated with mortality.

**Conclusions:**

MTB-BSI is far more common for inpatients than outpatients with HIV. Across multiple settings with high tuberculosis prevalence, MTB-BSI was strongly associated with heightened risk of early mortality in PWH. Novel optimized diagnostic and treatment strategies are still needed for HIV-associated MTB-BSI.

## Teaser

Despite scale-up of antiretrovirals, disseminated tuberculosis continues to be common in hospitalized patients with HIV and associated with heightened mortality risk. Optimized diagnostic and treatment strategies for people with HIV and disseminated tuberculosis are needed.

In people with HIV-1 (PWH), tuberculosis (TB) is the leading cause of death and hospitalization [1, 2]. In advanced HIV, TB is frequently extrapulmonary or disseminated, making it more difficult to diagnose and substantially increasing mortality risk [3–5]. The most effective tool to decrease the risk of TB disease and TB-related mortality in PWH is initiation or reinitiation of effective antiretroviral therapy (ART), which has been scaled up globally [5–8].

Disseminated TB in PWH can be diagnosed via (1) the detection of *Mycobacterium tuberculosis* (MTB) from >1 bodily site or (2) the detection of MTB bloodstream infection (MTB-BSI). Disseminated TB is also highly suggested when TB is diagnosed by urine-based tests such as a lipoarabinomannan (LAM) antigen assay (Determine-LAM; Abbott) or Xpert Ultra assay (Cepheid) [4, 9–12]. Mycobacterial blood culture is a key diagnostic tool for this patient group but has limited diagnostic sensitivity, particularly when only a single blood culture is performed [13].

A 2020 individual participant data meta-analysis of adults and adolescents with HIV found that MTB-BSI was common in hospitalized patients with HIV-associated TB (HIV-TB) [4]. The meta-analysis included 5751 participants from studies conducted between 1991 and 2017, spanning nearly 3 decades with substantial changes and scale-up of ART and TB prevention therapy. Among inpatients with HIV-TB who had abnormal vital signs and a median CD4 count of 76 cells/µL, 45% were predicted to have MTB-BSI if 2 blood cultures had been performed [4]. Risk of 30-day mortality was 2.5 times higher in those patients with MTB-BSI as compared with those with HIV-TB but without MTB-BSI, and this risk was even higher if TB treatment was delayed by as little as 4 days [4].

The current analysis is based on a modern cohort study of largely ART-experienced participants, in which we sought to assess whether MTB-BSI continues to be common and result in increased risk of early mortality among hospitalized and ambulatory adults from 5 countries in sub-Saharan Africa and 2 countries in Southeast Asia.

## METHODS

### Study Population

This multicounty prospective cohort study was nested within a diagnostic accuracy evaluation conducted between December 2019 and August 2021, with study methods described in detail elsewhere [14]. In brief, adults (≥18 years) with HIV were invited to enroll soon after admission to a medical ward or presentation to an outpatient clinic. Enrollment happened across 13 facilities in 7 countries with high TB burden in sub-Saharan Africa and Southeast Asia (Malawi, South Africa, Tanzania, Thailand, Uganda, Viet Nam, and Zambia). Certain countries recruited only inpatients (namely South Africa), certain countries recruited only outpatients (namely Tanzania), and the remainder recruited patients at inpatient and outpatient sites. Participants were recruited irrespective of their CD4 counts or ART status. Inpatients were also recruited irrespective of their symptoms, whereas outpatients were recruited only if they had at least 1 TB symptom (cough, fever, loss of weight, or night sweats). Exclusion criteria included having had >2 doses of anti-TB treatment within the prior 60 days, isoniazid preventive therapy within the prior 6 months, or an unwillingness or inability to provide informed consent to the study.

### Study Procedures

Demographics and clinical features were recorded on enrollment day. Participants also had 1 to 5 mL of blood drawn for mycobacterial culture and further blood for CD4 count by flow cytometry. Sputum was collected for Xpert Ultra and mycobacterial culture and urine for Xpert Ultra and Determine-LAM. When additional TB tests were performed by the routine clinical team within 3 months of enrollment, such as lymph node aspirates or cerebrospinal fluid but including additional blood sent for mycobacterial culture, these were also captured into the study database. The diagnosis of TB was made via a positive Xpert Ultra or mycobacterial culture from any clinical site. Vital status was recorded up to 70 days per the original study protocol, via in-person visits, telephone, or checks of electronic health records [14].

Blood for mycobacterial culture was collected directly into culture vials (BACTEC Myco/F Lytic; Becton Dickinson) with automated monitoring for positivity over ≥42 days and with mycobacterial speciation of a positive result done with MPT64 antigen detection and/or MTBDR*plus*, MTBC, and CM/AS line probe assays (Hain Lifescience) per local standards (Supplementary Table 1).

### Ethics

Each site obtained approval from its local research ethics committee before initiation of any study procedures [14]. Written informed consent was obtained from all participants in their preferred language.

### Statistical Analysis

We summarized characteristics of study participants and compared them by MTB-BSI status. We calculated the crude prevalence of MTB-BSI as the number with positive MTB-BSI results over the number with valid results (ie, positive or negative), in inpatients and outpatients overall, as well as within the inpatients and outpatients diagnosed with microbiologically confirmed TB. Subsequently, we constructed Bayesian multilevel logistic regression models to investigate factors associated with prevalent MTB-BSI in the same patient groups. We included a random effects term for study country and fixed effects terms for age, CD4 count, and their interaction. Priors were weakly informative, and model fitting was checked by inspecting trace plots, Gelman-Rubin statistics, and effective sample sizes. We took 4000 draws from the posterior and summarized by mean and quantile-based 95% credible interval (95% CrI) to estimate overall and country-specific prevalence as well as odds ratios for the association between covariates and prevalence.

We investigated the risk of MTB-BSI on the hazard of death for inpatients only, overall and within the inpatients with microbiologically confirmed TB, using Bayesian regression models with proportional hazards distributional families. The primary outcomes were hazard of 30- and 70-day mortality from MTB-BSI (confirmed via mycobacterial blood culture) in adults hospitalized with HIV. The 70-day endpoint was per the primary diagnostic accuracy study. The 30-day outcome was added as a co–primary assessment of early mortality. We investigated variation in hazard of death for inpatients among countries by including random intercept and slope terms, and we modeled predicted hazard ratios for age and CD4 count by including an interaction term. Secondary outcomes included the association between covariates (age, CD4 count, and ART status) and hazard of death. We also explored the effect of region (Africa vs Asia) on the hazards of mortality with a fixed effects term. In prespecified sensitivity analysis—recognizing the imperfect sensitivity of mycobacterial blood cultures—we estimated the hazard of 70-day mortality from MTB-BSI in adults hospitalized with HIV, where disseminated TB was confirmed via mycobacterial blood culture or when the patient had positive urine results from both Xpert Ultra and Determine-LAM. In a post hoc sensitivity analysis, we explored the potential effect of the group lost to follow-up and whether it influenced our conclusions, by estimating the hazards of death at a probability of 0% to 100%, presuming that those lost to follow-up had died. We also compared Bayesian estimates against those obtained from frequentist Cox proportional hazard models, and our weakly informative prior against that of more informative priors [4] from the literature. All analysis was done in R version 4.50 with Bayesian models fit via the rstanarm interface to Stan [15].

The study protocol's primary objectives were to assess the diagnostic accuracy of the first-generation Fujifilm SILVAMP TB LAM (FujiLAM I). Sample size calculations were thereby based on the expected diagnostic accuracy of FujiLAM I; the sample size calculations and results of which are reported elsewhere [14]. No post hoc sample size calculations were done for this analysis. Reporting followed the STROBE guidelines for cohort studies (for checklist, see Supplementary material).

## RESULTS

### Participant Characteristics

**Figure 1.**
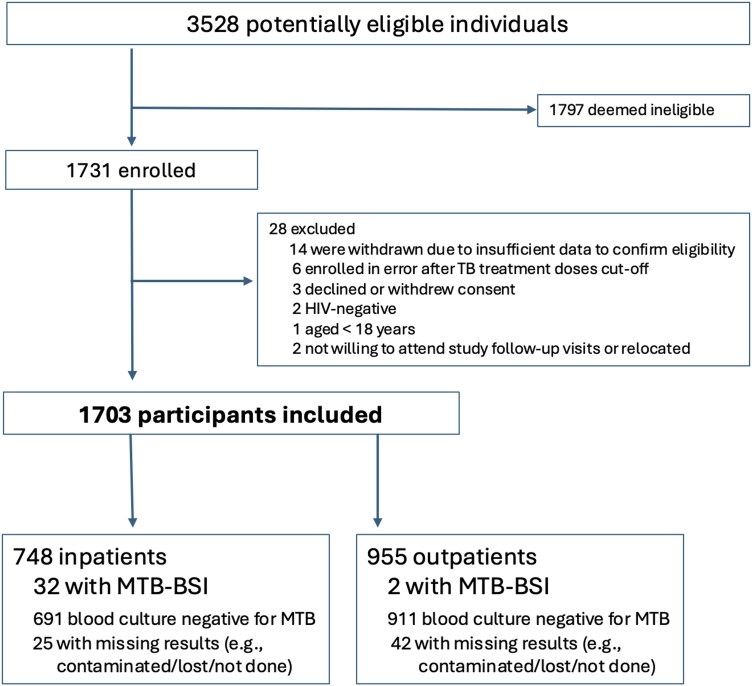
Participant flow diagram. MTB-BSI, *Mycobacterium tuberculosis* bloodstream infection; TB, tuberculosis.

In total, 1703 adults were included in this analysis, of whom 748 (43.9%) were hospitalized and 955 (56.1%) were outpatients (Figure 1). Participants had a median age of 40 years and 52% were female. The cohort had a median CD4 count of 361 cells/μL, with 32.7% (557/1703) having a CD4 count ≤200 cells/μL. HIV viral loads were not performed by the study, but 77.5% (1319/1703) of the participants reported being prescribed ART at enrollment. Of the 1703 participants, 1687 (99.1%) had blood sent for mycobacterial blood culture. Contamination rates of the mycobacterial blood cultures per country varied between 0% and 9.9%, with 6 of 7 countries having <5% contamination rate (Supplementary Table 2).

### Prevalence of MTB-BSI

Crude prevalence of MTB-BSI was 4.4% in overall inpatients (32/723) and 22.5% (32/142) among inpatients with microbiologically confirmed TB. The crude prevalence of MTB-BSI was 0.2% in overall outpatients (2/913) and 1.4% (2/139) among outpatients with microbiologically confirmed TB. MTB-BSI prevalence varied by country with the highest prevalence being in South Africa (23/195, 11.8%), followed by Viet Nam (3/167, 1.8%; Supplementary Table 2). Notably, South Africa was the only study site to recruit exclusively inpatients, and across all inpatient cohorts in the study countries, South African inpatients had the lowest median CD4 count (111 cells/μL). Participants who had confirmed MTB-BSI, as compared with those without MTB-BSI, were generally younger and had lower CD4 counts (Table 1, Supplementary Tables 3 and 4). In those with MTB-BSI, 32% reported being prescribed ART, as opposed to 79% of those without MTB-BSI. In the group with microbiologically confirmed TB at any site, the median CD4 count was 183 cells/μL. Two participants (0.1% of overall cohort) had nontuberculous mycobacteria identified on blood culture. In the overall cohort, 42 participants had positive urine results by both Xpert Ultra and Determine-LAM, but their mycobacterial blood cultures were negative; as such, they represented a group of patients with disseminated TB who may have had MTB-BSI that was missed by the imperfect sensitivity of a single blood culture (Supplementary Figure 1).

**Table 1.** Patient Demographics and Clinical Characteristics per MTB-BSI Status

	MTB-BSI, No. (%) or Median (IQR)			
	Overall (N = 1703)	Positive (n = 34)	Negative (n = 1602)	Unclassifiable ^a^ (n = 67)
Site				
Malawi	349	3 (0.9)	344 (98.6)	2 (0.6)
South Africa	197	23 (11.7)	172 (87.3)	2 (1.0)
Tanzania	242	0	218 (90.1)	24 (9.9)
Thailand	131	1 (0.8)	129 (98.5)	1 (0.8)
Uganda	247	4 (1.6)	235 (95.1)	8 (3.2)
Viet Nam	177	3 (1.7)	164 (92.7)	10 (5.6)
Zambia	360	0	340 (94.4)	20 (5.6)
Demographics				
Age, y	40 (33–48)	35 (27–41)	41 (32–48)	40 (34–46)
Female	894	20 (2.2)	842 (94.2)	32 (3.6)
ART status at presentation				
Current use	1319	11 (0.8)	1260 (95.5)	48 (3.6)
Past use	99	12 (12.1)	83 (83.8)	4 (4.0)
Naive	267	11 (4.1)	243 (91.0)	13 (4.9)
Status unknown	18	0	16 (88.9)	2 (11.1)
Clinical feature at presentation				
Known TB history	449	10 (2.2)	417 (92.9)	22 (4.9)
Seriously ill criteria ^b^	754	23 (3.1)	698 (92.6)	33 (4.4)
CD4 count	361 (126–624)	35 (12–103)	372 (140–629)	260 (80–560)
Vital status outcome at 70 d				
Alive	1454	25 (1.7)	1376 (94.6)	53 (3.6)
Died	137	9 (6.7)	121 (88.3)	7 (5.1)
Lost to follow-up	112	0	105 (93.8)	7 (6.3)

Abbreviations: ART, antiretroviral therapy; MTB-BSI, *Mycobacterium tuberculosis* bloodstream infection; TB, tuberculosis.

^a^Unclassifiable due to a contaminated, lost, missing, or unknown mycobacterial blood culture result.

^b^Defined as having any of the following: body mass index <18.5 kg/m^2^, respiratory rate >30 breaths/min, systolic blood pressure <90 mm Hg, heart rate >120 beats/min, or the inability to walk unaided.

**Figure 2.**
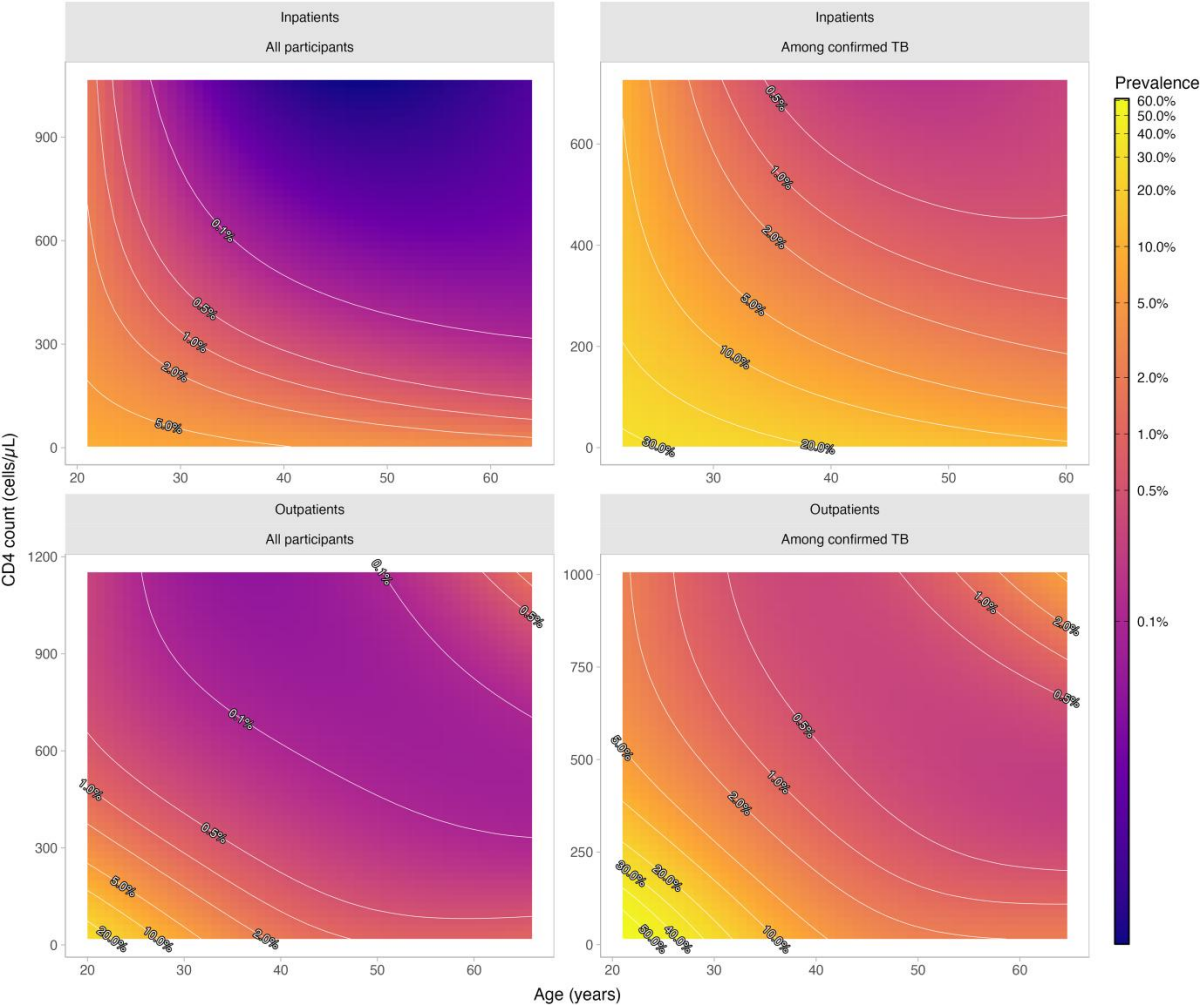
Predicted prevalence of *Mycobacterium tuberculosis* bloodstream infection by age and CD4 count for inpatients (top) and outpatients (bottom) and within all (left) and within those who had microbiologically confirmed tuberculosis (TB; right).

In Bayesian regression models, the estimated prevalence of MTB-BSI among all inpatients (n = 647) was 5.0% (95% CrI, 3.4%–6.8%), and that among inpatients who had microbiologically confirmed TB (n = 133) was 24.2% (95% CrI, 17.5%–31.4%). Among all outpatients (n = 882), prevalence was 0.3% (95% CrI, .1%–.8%) as opposed to 2.0% (95% CrI, .4%–4.7%) for outpatients who had microbiologically confirmed TB (n = 135). Prevalence was higher in those with lower CD4 counts and younger age (Figure 2) and differed by country (Supplementary Figure 2).

### Diagnosis and Treatment of MTB-BSI

Of the 34 participants (inpatients or outpatients) with confirmed MTB-BSI, 24 (70.6%) had a positive urine result by Xpert Ultra, 18 (52.9%) had a positive sputum result by Xpert Ultra, and 20 (58.8%) had a positive urine result by Determine-LAM. Moreover, 29 participants (85.3%) were positive by at least 1 of these rapid tests, leaving 14.7% needing to rely on delayed culture results. In the group with HIV-TB but no MTB-BSI, a similar percentage (83.3%, 219/263) was positive by any of these 3 rapid tests for TB (urine Xpert Ultra, 35.7%; sputum Xpert Ultra, 63.9%; urine Determine-LAM, 26.2%).

In the group with MTB-BSI, 76.5% (26/34) had initiated TB treatment within 3 days of enrollment to this study. In the group with HIV-TB but no MTB-BSI, early TB treatment initiation was less common: 44.9% (118/263) started TB treatment within 3 days of enrollment. Of those with MTB-BSI who did not receive treatment initiation within 3 days of enrollment, 2 initiated treatment later, 1 died before treatment initiation, and 5 were not known to have initiated TB treatment by the study teams.

### Mortality due to MTB-BSI

**Figure 3.**
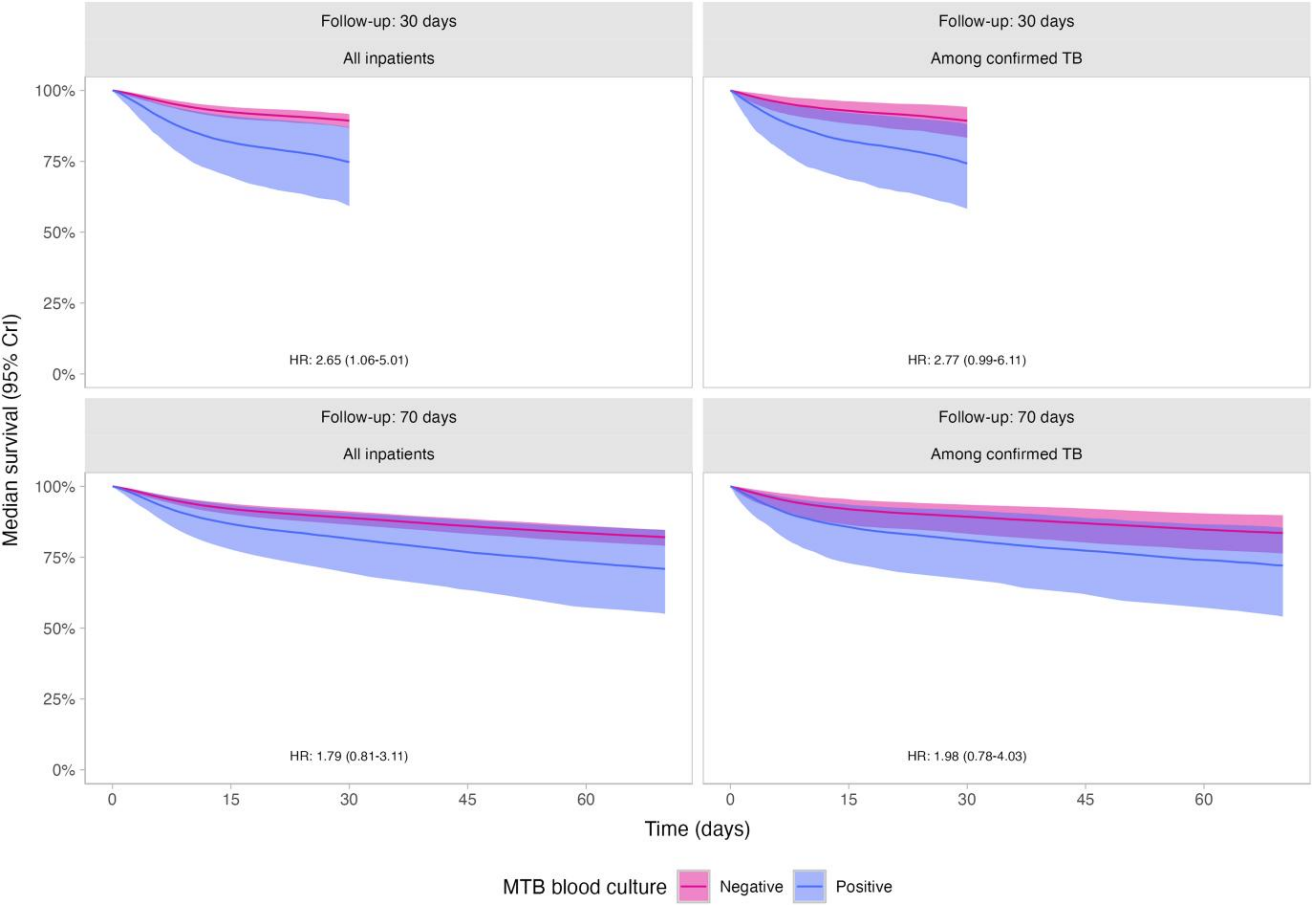
Mortality among inpatients by positive vs negative result for MTB-BSI. An unadjusted model predicted survival up to 30 days (top) and 70 days (bottom) of follow-up in the overall group (left) and within those who had microbiologically confirmed TB (right). 95% CrI, 95% credible interval; HR, hazard ratio; MTB-BSI, *Mycobacterium tuberculosis* bloodstream infection; TB, tuberculosis.

**Figure 4.**
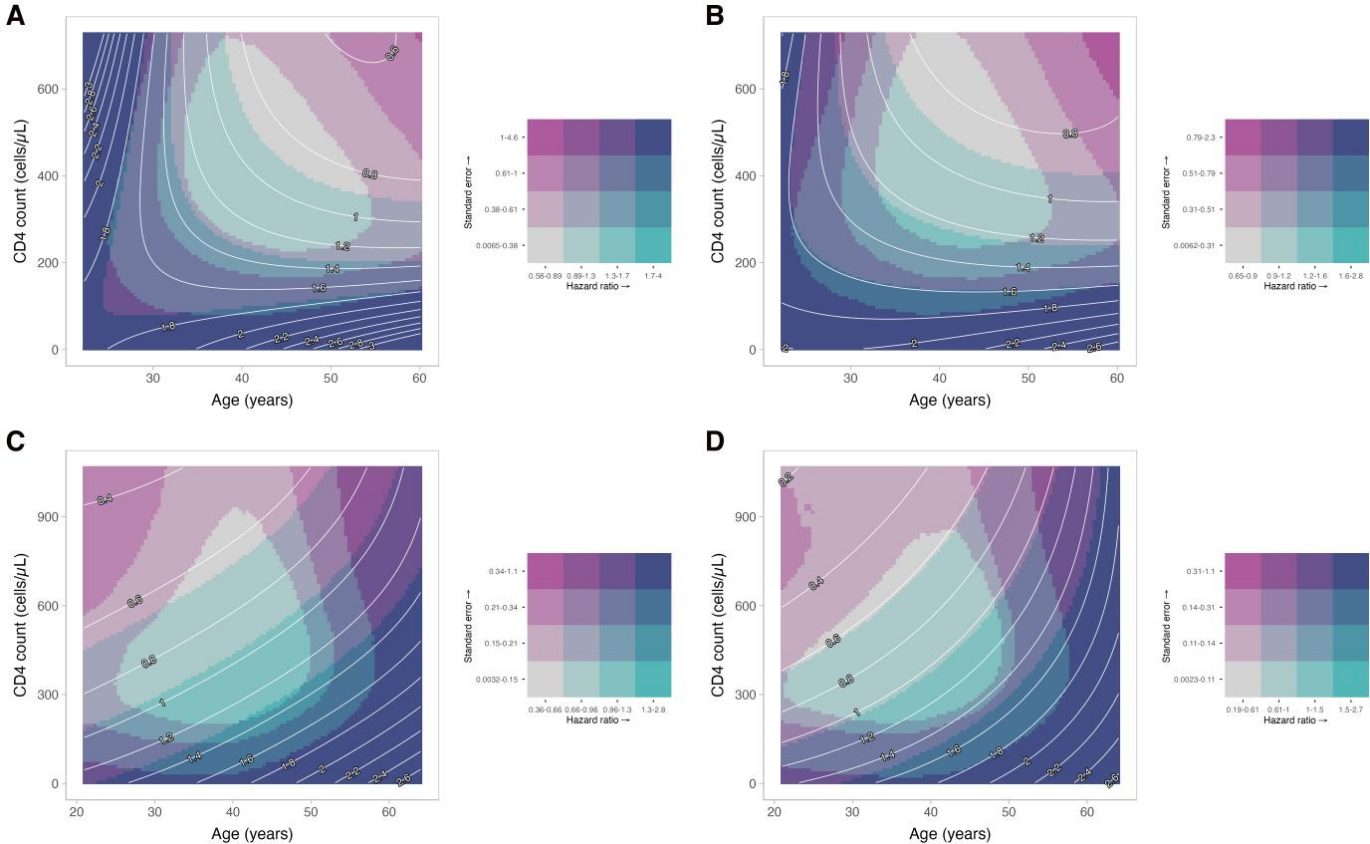
The hazard of death by age and CD4 count: *A*, all inpatients to 30 days; *B*, inpatients who had microbiologically confirmed TB to 30 days; *C*, all inpatients to 70 days; *D*, inpatients who had microbiologically confirmed TB to 70 days. TB, tuberculosis.

It was not possible to estimate the hazards of mortality from MTB-BSI in outpatients due to the low prevalence of MTB-BSI (2 participants). Both participants were alive at the end of our study follow-up period. Primary analysis focused therefore on inpatients. Inpatient participants with MTB-BSI confirmed by mycobacterial blood culture had a 2.65-times greater hazard of death by 30 days (95% CrI, 1.06–5.01) and 1.79-times greater hazard of death by 70 days (95% CrI, .81–3.11; Figure 3). Participants with lower CD4 counts and older age were at substantially increased hazard of death (Figure 4). Participants who reported being prescribed ART at enrollment vs not or having an unknown ART status had a hazard ratio for 30-day mortality of 0.84 (95% CrI, .41–1.36). There was evidence of a difference by region, with the hazard of mortality by day 30 being 1.97 (95% CrI, 1.17–3.19) for Africa vs Asia. For inpatients with microbiologically confirmed TB with or without MTB-BSI, having MTB-BSI resulted in a 2.77-times greater hazard of death by 30 days (95% CrI, .99–6.11) and 1.98-times greater hazard of death by 70 days (95% CrI, .78–4.03).

In a sensitivity analysis with the expanded case definition—including a positive mycobacterial blood culture or a positive urine results by both Xpert Ultra and Determine-LAM—the hazard ratio for mortality by 30 days was 1.91 (95% CrI, .91–3.43) in overall inpatients and 2.15 (95% CrI, .75–4.90) for inpatients with microbiologically confirmed TB. At 70 days, this hazard ratio was 1.59 (95% CrI, .86–2.55) in overall inpatients and 2.08 (95% CrI, .89–4.26) for inpatients with microbiologically confirmed TB (Supplementary Figure 3).

While the proportion of participants who were lost to follow-up at 70 days was <10%, it was more common in the group without MTB-BSI (6.6%) than that with confirmed MTB-BSI (0%). We therefore performed sensitivity analysis to assess the impact on hazard ratios for mortality according to the fraction being lost to follow-up and the probability of death in that group. When we assumed that the group lost to follow-up had died at a range of 0% to 100%, our estimated hazard ratios were similar, and all had overlapping credible intervals (Supplementary Figure 4). When more informative priors were used, the hazard ratio for 30-day mortality among inpatients was 2.55 (95% CrI, 1.36–4.54). Hazard ratios were also similar when we compared estimates from Bayesian and frequentist models (Supplementary Figure 5).

## DISCUSSION

MTB-BSI continues to be common in hospitalized PWH and is associated with heightened mortality risk, even in the modern era with high rates of ART. In this multinational cohort from sub-Saharan Africa and Southeast Asia with a median CD4 count of 361 cells/μL, approximately 1 in 20 PWH admitted to the hospital had MTB-BSI irrespective of symptoms, and within the subgroup of inpatients with HIV-TB, 24.2% had MTB-BSI. While MTB-BSI is far less common in ambulatory PWH, it still occurs. MTB-BSI is associated with a higher risk of mortality, particularly within the first 30 days after admission.

Currently, MTB-BSI is managed in the same way as pulmonary TB, wherein 2 months of rifampicin, isoniazid, ethambutol, and pyrazinamide is given, followed by another 4 months of rifampicin and isoniazid (for rifampicin-sensitive MTB). Based on prior evidence that patients hospitalized with HIV-TB were more likely to have died if they demonstrated higher bacillary loads [9, 16] or increased markers of innate immune activation [16], 3 randomized controlled trials are assessing whether intensified TB treatment with or without a course of corticosteroids can improve survival among inpatients with HIV-TB [17, 18]. Access to the point-of-care urine Determine-LAM assay is associated with improved survival in ill inpatients with HIV [19, 20]. Initiating TB treatment at hospital presentation for PWH who had features of sepsis decreased 28-day mortality for the subgroup that was later confirmed to have TB [21]. Avoiding diagnostic and treatment delays is thereby also key to the management of this group [4]. Patients with MTB-BSI in this cohort had high rates of TB treatment initiation within the first 3 days of study enrollment.

In a meta-analysis of MTB-BSI prevalence in cohorts spanning 1991 to 2017, the average inpatient diagnosed with TB at any site had an approximately 45% probability of having MTB-BSI [4]. In a meta-analysis of causes of hospital admissions between 2014 and 2023, TB at any site was the leading cause of hospitalization (19%) [2]. In recent outpatient cohorts of advanced HIV or PWH without ART, urine tests were able to detect TB, suggesting a disseminated component of disease, although mycobacterial blood cultures are generally not performed in outpatient settings [22, 23].

Our cohort adds data from 748 inpatients and 955 outpatients to the prior evidence and shows that MTB-BSI, a severe subgroup of TB, continues to be common—estimated at 5.0% of PWH admitted to hospital, irrespective of their symptoms, or 24.2% within inpatient PWH who had microbiologically confirmed TB at any site. It is important to note that this varied by country. The decreased estimate from 45% (95% CI, 38%–52%) in the prior MTB-BSI meta-analysis to 24% (95% CrI, 18%–31%) in the current study could be due to differences in these estimate assumptions; for example, the former assumed that 2 blood cultures were done per participant, supporting greater diagnostic yield for MTB-BSI, whereas our study protocol included only 1 blood culture. Furthermore, the meta-analysis included more patients with more severe immunosuppression (median CD4, 76 cells/μL) as compared with our cohort (median CD4, 361 cells/μL overall or 182 cells/μL for inpatients).

The high rates of early treatment initiation may have further negatively affected the diagnostic yield of mycobacterial blood cultures in our cohort. While we did not perform HIV viral loads in this study, a large proportion of participants reported being prescribed ART at enrollment, but this was less common in participants with MTB-BSI. It is possible that part of this difference in prevalence estimate is due to greater coverage with effective ART leading to less severe immunosuppression. The prior meta-analysis found that in cases of TB with MTB-BSI vs those without, the hazard of death within 1 month was 2.48 [4], and we have shown that this increased hazard does not appear to be changing, as estimated in this cohort at 2.77 within 1 month. While estimates of MTB-BSI prevalence for inpatients with HIV-TB might be decreasing, this is still very common and continues to cause heightened risk of early mortality. Other more recent cohorts have noted crude estimates of MTB-BSI prevalence that are more similar to ours [21], but an updated meta-analysis would be required to assess whether this prevalence is decreasing.

While our study was conducted in multiple African and Asian health care facilities, these results cannot be generalized to settings with low TB burden. Similarly, in settings with high TB burden, our results cannot necessarily be generalized to more underresourced or rural areas, where there might be less access to ART as compared with our study hospitals and clinics.

There are important limitations to consider in this study. First, MTB-BSI prevalence was estimated from a single blood culture in the majority of the cohort, despite there being evidence that this might miss cases of true MTB-BSI [13]. While a standard volume of blood was added to each vial, we also did not capture data on the volume of blood collected per participant. To overcome the potential diagnostic yield limitations of mycobacterial blood culture, we performed a sensitivity analysis with urine-based tests that are recognized to have good diagnostic yield for detecting disseminated TB [4, 9]. Second, approximately 6.6% of participants had an unknown vital status at 70 days, and notably this occurred only in the group that was negative for MTB-BSI rather than positive. We performed a sensitivity analysis to recognize the potential effects of this finding on our interpretation. Third, this cohort was enrolled from 2019 to 2021, and our estimates might have been affected by treatment delays due to the COVID-19 pandemic and patients thereby presenting to care with more severe illness. Importantly, this study did not measure HIV viral loads at enrollment, and self-reporting of ART status risks misclassification. Our modeled estimates showed that immunosuppression had a strong impact on mortality, whereas self-reported ART status did not show strong evidence of a difference in outcome. CD4 counts were objectively assessed rather than based on self-reported ART status. While it is likely that MTB-BSI is primarily occurring in those not undergoing effective ART, it would have been preferrable to measure this objectively with HIV viral loads.

MTB-BSI continues to be common and causes heightened risk of death among inpatients with HIV in the modern ART era. We expect authors of various randomized controlled trials of optimized treatment strategies for inpatients with HIV-TB to publish their data during 2026. Strategies that focus on ART scale-up and retention to prevent MTB-BSI, as well as improved diagnostic tests or approaches that can support treatment initiation soon after hospital presentation, are urgently needed to decrease deaths from TB.

## Supplementary Material

ofag424_Supplementary_Data

## Supplementary Data

Supplementary materials are available at *Open Forum Infectious Diseases* online. Consisting of data provided by the authors to benefit the reader, the posted materials are not copyedited and are the sole responsibility of the authors, so questions or comments should be addressed to the corresponding author.

## Notes

***Acknowledgments.*** The authors thank the patients, their families, and the clinical teams at the study sites for their contribution to this cohort.

***Data availability.*** Data are available upon reasonable request to the corresponding author.

***Disclaimer.*** The views expressed are those of the authors and not necessarily those of the NIHR or the Department of Health and Social Care. The funders had no role in the study design, analysis or decision to publish results.

***Open access.*** For the purpose of open access, the authors have applied a CC BY public copyright licence to any author accepted manuscript version arising from this submission.

***Financial support.*** This work was supported by the Global Health Innovative Technology Fund (G2017-207); the KfW Development Bank (2020 60 457); the Commonwealth of Australia represented by the Department of Foreign Affairs and Trade (70957); the Netherlands Enterprise Agency (PDP15CH14); Wellcome (304666/Z/23/Z to P. M.); an NIHR Global Health Research Professorship (NIHR304311 to P. M.); the Fogarty International Center of the National Institutes of Health and the Eunice Kennedy Shriver National Institute of Child Health & Human Development (D43 TW010559 to B. S.); the Wellcome Trust (214321/Z/18/Z and 203135/Z/16/Z to G. M.); and the South African Research Chairs Initiative of the Department of Science and Technology and National Research Foundation of South Africa (grant 64787 to G. M.).

***Potential conflicts of interest.*** R. S., C. M. D., and M. R. were employed by FIND, the global alliance for diagnostics, at the time of the study. FIND is a not-for-profit foundation that supports the evaluation of publicly prioritized tuberculosis assays and the implementation of World Health Organization–approved assays (guidance and prequalification) using donor grants. FIND has product evaluation agreements with several private sector companies that design diagnostics for tuberculosis and other diseases; these agreements strictly define FIND's independence and neutrality with regard to these private sector companies. All other authors report no potential conflicts.
