# Supplementary material for: Prevalence and Mortality due to Mycobacterium tuberculosis Bloodstream Infection in Adults With HIV: A Multicountry Prospective Cohort Study
Source: Open Forum Infect Dis. 2026 Jul 7;13(7):ofag424. doi: 10.1093/ofid/ofag424 (PMC13403080; doi:10.1093/ofid/ofag424)
Supplement: ofag424_Supplementary_Data [file ofag424_supplementary_data.pdf]

Supplementary Material

Prevalence and mortality due to *Mycobacterium tuberculosis* bloodstream infection in adults with HIV: a multi-country prospective cohort study

Authors

Bianca Sossen, Madalo Mukoka, Rita Székely, Monde Muyoyeta, Elizabeth Nakabugo, Jerry Hella, Hung Van Nguyen, Sasiwimol Ubolyam, Marcia Vermeulen, Chad M Centner, Sarah Nyangu, Nsala Sanjase, Mohamed Sasamalo, Huong Thi Dinh, The Anh Ngo, Weerawat Manosuthi, Supunnee Jirajariyavej, Nhung Viet Nguyen, Anchalee Avihingsanon, Claudia M Denkinger, Klaus Reither, Lydia Nakiyingi, Andrew D Kerkhoff, Morten Ruhwald, Graeme Meintjes, and Peter MacPherson

Table of Contents

Supplementary table 1: Laboratory procedures per country for study mycobacterial blood cultures 2

STROBE Statement—Checklist of items that should be included in reports of ***cohort studies*** 3

Supplementary table 2: Mycobacterial blood culture results by country and setting 6

Supplementary table 3: Demographic and clinical characteristics, per MTB-BSI status in inpatients only......7

Supplementary table 4: Demographic and clinical characteristics, per MTB-BSI status in outpatients only ...8

Supplementary Figure 1: Euler plot of participants positive by Mycobacterial blood culture (MTB-BC), urine Xpert Ultra (UrineXPU) and urine Determine-LAM in relation to participants confirmed by any other test for MTB[a]......9

Supplementary Figure 2: Predicted prevalence of *Mycobacterium tuberculosis* bloodstream infection (MTB-BSI) by country in inpatients (top) and outpatients (bottom) and within all (left) or within those who had microbiologically confirmed TB (right). 10

Supplementary Figure 3: Survival in sensitivity analysis with the expanded case definition of having either a mycobacterial blood culture positive for MTB, or having both urine Xpert Ultra and Determine-LAM tests positive 11

Supplementary figure 4: Sensitivity analysis of hazards of mortality according to the probability of death amongst those who were lost to follow up 12

Supplementary Figure 5: Comparison of estimates from Bayesian and frequentist models in inpatients, with hazard ratios on the X axis and model structure on the Y axis......13

Supplementary table 1: laboratory procedures per country for study mycobacterial blood cultures

Country	Collection vials	Monitoring for positivity	Speciation	Duration of incubation to deem negative
Malawi	BACTEC™	Automated	MPT64	42 days
South Africa	BACTEC™	Automated	MPT64 antigen and line probe assay	42 days
Tanzania	BACTEC™	Automated	Line probe assay	42 days
Thailand	BACTEC™	Automated	MPT64 antigen	42 days
Uganda	BACTEC™	Automated	MPT64 antigen	56 days
Viet Nam	BACTEC™	Automated	MTP64 antigen	42 days
Zambia	BACTEC™	Automated	MPT64 antigen	42 days

STROBE Statement—Checklist of items that should be included in reports of ***cohort studies***

	Item No	Recommendation	Page No
**Title and abstract**	1	(*a*) Indicate the study's design with a commonly used term in the title or the abstract	1
		(*b*) Provide in the abstract an informative and balanced summary of what was done and what was found	3
**Introduction**			
Background/rationale	2	Explain the scientific background and rationale for the investigation being reported	4-6
Objectives	3	State specific objectives, including any prespecified hypotheses	5-6
**Methods**			
Study design	4	Present key elements of study design early in the paper	5-8
Setting	5	Describe the setting, locations, and relevant dates, including periods of recruitment, exposure, follow-up, and data collection	5-8
Participants	6	(*a*) Give the eligibility criteria, and the sources and methods of selection of participants. Describe methods of follow-up	5-6
		(*b*) For matched studies, give matching criteria and number of exposed and unexposed	N/A
Variables	7	Clearly define all outcomes, exposures, predictors, potential confounders, and effect modifiers. Give diagnostic criteria, if applicable	6-8
Data sources/ measurement	8*	For each variable of interest, give sources of data and details of methods of assessment (measurement). Describe comparability of assessment methods if there is more than one group	6-8
Bias	9	Describe any efforts to address potential sources of bias	8
Study size	10	Explain how the study size was arrived at	8
Quantitative variables	11	Explain how quantitative variables were handled in the analyses. If applicable, describe which groupings were chosen and why	7-8
Statistical methods	12	(*a*) Describe all statistical methods, including those used to control for confounding	7-8

		(*b*) Describe any methods used to examine subgroups and interactions	7-8
		(*c*) Explain how missing data were addressed	7-8
		(*d*) If applicable, explain how loss to follow-up was addressed	8
		(*e*) Describe any sensitivity analyses	7-8
**Results**			
Participants	13*	(a) Report numbers of individuals at each stage of study—eg numbers potentially eligible, examined for eligibility, confirmed eligible, included in the study, completing follow-up, and analysed	9-10
		(b) Give reasons for non-participation at each stage	Fig1
		(c) Consider use of a flow diagram	Fig1
Descriptive data	14*	(a) Give characteristics of study participants (eg demographic, clinical, social) and information on exposures and potential confounders	Table1
		(b) Indicate number of participants with missing data for each variable of interest	Table1
		(c) Summarise follow-up time (eg, average and total amount)	Table1, supplementary figure 4
Outcome data	15*	Report numbers of outcome events or summary measures over time	Figure 4<br>P15-17

Main results	16	(*a*) Give unadjusted estimates and, if applicable, confounder-adjusted estimates and their precision (eg, 95% confidence interval). Make clear which confounders were adjusted for and why they were included	15-17
		(*b*) Report category boundaries when continuous variables were categorized	N/A
		(*c*) If relevant, consider translating estimates of relative risk into absolute risk for a meaningful time period	N/A
Other analyses	17	Report other analyses done—eg analyses of subgroups and interactions, and sensitivity analyses	15-17
**Discussion**			
Key results	18	Summarise key results with reference to study objectives	17-18
Limitations	19	Discuss limitations of the study, taking into account sources of potential bias or imprecision. Discuss both direction and magnitude of any potential bias	19-20
Interpretation	20	Give a cautious overall interpretation of results considering objectives, limitations, multiplicity of analyses, results from similar studies, and other relevant evidence	17-20
Generalisability	21	Discuss the generalisability (external validity) of the study results	19
**Other information**			
Funding	22	Give the source of funding and the role of the funders for the present study and, if applicable, for the original study on which the present article is based	21

*Give information separately for exposed and unexposed groups.

Supplementary table 2: mycobacterial blood culture results by country and setting

		Malawi (n=349)	South Africa (n=197)	Tanzania (n=242)	Thailand (n=131)	Uganda (n=247)	Viet Nam (n=177)	Zambia (n=360)	**Inpatients (n=748)**	**Outpatients (n=955)**	**Overall (n=1703)**
Recruitment setting	Inpatients	174	197	0	31	125	78	143	**748**	**0**	**748**
	Outpatients	175	0	242	100	122	99	217	**0**	**955**	**955**
Study blood culture	No growth of MTB	344	178	218 (2 grew NTM)	129	235	164	340	**691**	**911 (including 2 grew NTM)**	**1608 (2 grew NTM)**
	Growth of MTB complex	3 (0.9%)	17 (8.6%)	0	1 (0.8%)	4 (1.6%)	3 (1.7%)	0	**26**	**2**	**28**
	Contaminated / lost	0 (0%)	1 (0.5%)	24 (9.9%)	0 (0%)	7 (2.8%)	6 (3.4%)	12 (3.3%)	**15**	**35**	**50**
	Result not captured or blood culture not done	2	1	0	1	1	4	8	**10**	**7**	**17**
Clinical team blood culture	Growth of MTB complex, where a study blood culture not positive (i.e., additional positive blood cultures from the clinical team's tests)	0	6	0	0	0	0	0	**6**	**0**	**6**

Supplementary table 3: demographic and clinical characteristics, per MTB-BSI status in inpatients only

	Overall (n=748)	**MTB-BSI positive (n=32)**	**MTB-BSI negative (n=691)**	MTB-BSI unclassifiable[a] (n=25)
Site; n (%)				
Malawi	174	2 (0.1)	170 (97.7)	2 (0.1)
South Africa	197	23 (11.7)	172 (87.3)	2 (0.1)
Thailand	31	0	31 (100)	0
Uganda	125	4 (3.2)	115 (92.0)	6 (4.8)
Viet Nam	78	3 (3.8)	67 (85.9)	8 (10.3)
Zambia	143	0	136 (95.1)	7 (4.9)
Demographics				
Age, years; median (IQR)	39 (32 – 47)	35 (28 – 42)	40 (32 – 47)	39 (34 – 49)
Female; n (%)	392	19 (4.8)	362 (92.3)	11 (2.8)
ART status at presentation; n (%)				
Currently on ART	511	11 (2.2)	487 (95.3)	13 (2.5)
ART in the past	82	11 (13.4)	67 (81.7)	4 (4.9)
ART naïve	142	10 (7.0)	126 (88.7)	6 (4.2)
ART status unknown	13	0	11 (84.6)	2 (15.4)
Clinical features at presentation				
Known TB history; n (%)	193	10 (5.2)	175 (90.7)	8 (4.1)
Seriously ill criteria[c]; n (%)	435	22 (5.1)	393 (90.3)	20 (4.6)
CD4 count; median (IQR)	182 (55 – 408)	35 (11 – 95)	208 (63 – 432)	64 (22 – 267)
70-day vital status outcome; n (%)				
Alive	540	23 (4.3)	504 (93.3)	13 (2.4)
Died	127	9 (7.1)	111 (87.4)	7 (5.5)
Lost to follow up	81	0	76 (93.8)	5 (6.2)

Supplementary table 4: demographic and clinical characteristics, per MTB-BSI status in outpatients only

	Overall (n=955)	**MTB-BSI positive (n=2)**	**MTB-BSI negative (n=911)**	MTB-BSI unclassifiable[a] (n=42)
Site; n (%)				
Malawi	175	1 (0.6)	174 (99.4)	0
Tanzania	242	0	218 (90.1)	24 (9.9)
Thailand	100	1 (1.0)	98 (98.0)	1 (1.0)
Uganda	122	0	120 (98.4)	2 (1.6)
Viet Nam	99	0	97 (98.0)	2 (2.0)
Zambia	217	0	204 (94.0)	13 (6.0)
Demographics				
Age, years; median (IQR)	41 (34 – 49)	25 (22 – 28)	41 (34 – 49)	40 (34 – 44)
Female; n (%)	502	1 (0.2)	479 (95.4)	21 (4.2)
ART status at presentation; n (%)				
Currently on ART	808	0	773 (95.7)	35 (4.3)
ART in the past	17	1 (5.9)	16 (94.1)	0
ART naïve	125	1 (0.8)	117 (93.6)	0
ART status unknown	5	0	5 (100)	0
Clinical features at presentation				
Known TB history; n (%)	256	0	242 (94.5)	14 (5.5)
Seriously ill criteria[c]; n (%)	319	1 (0.3)	305 (95.6)	13 (4.1)
CD4 count; median (IQR)	497 (267 – 701)	85 (50 – 119)	500 (272 – 705)	366 (186 – 621)
70-day vital status outcome; n (%)				
Alive	914	2 (0.2)	872 (95.4)	40 (4.4)
Died	10	0	10 (100)	0
Lost to follow up	31	0	29 (93.5)	2 (6.5)

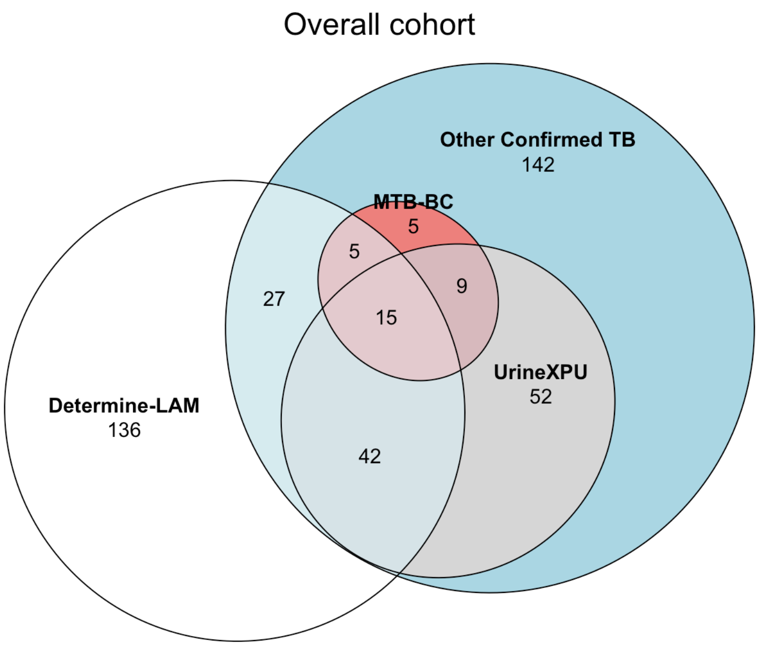

Supplementary Figure 1: Euler plot of participants positive by Mycobacterial blood culture (MTB-BC), urine Xpert Ultra (UrineXPU) and urine Determine-LAM in relation to participants confirmed by any other test for MTB[a]

a Tests for MTB including Xpert Ultra or mycobacterial culture on any sample

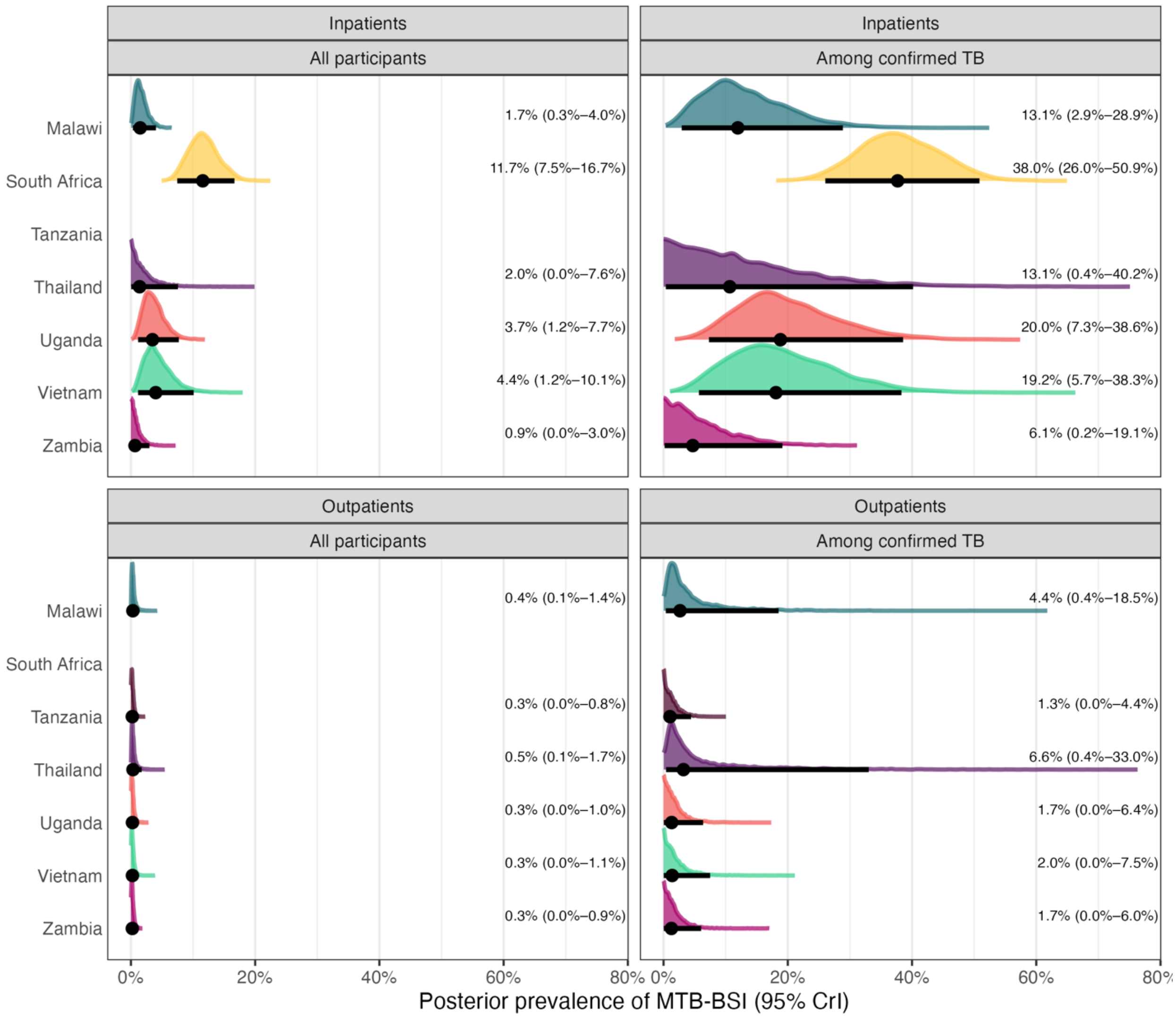

Supplementary Figure 2: Predicted prevalence of *Mycobacterium tuberculosis* bloodstream infection (MTB-BSI) by country in inpatients (top) and outpatients (bottom) and within all (left) or within those who had microbiologically confirmed TB (right).

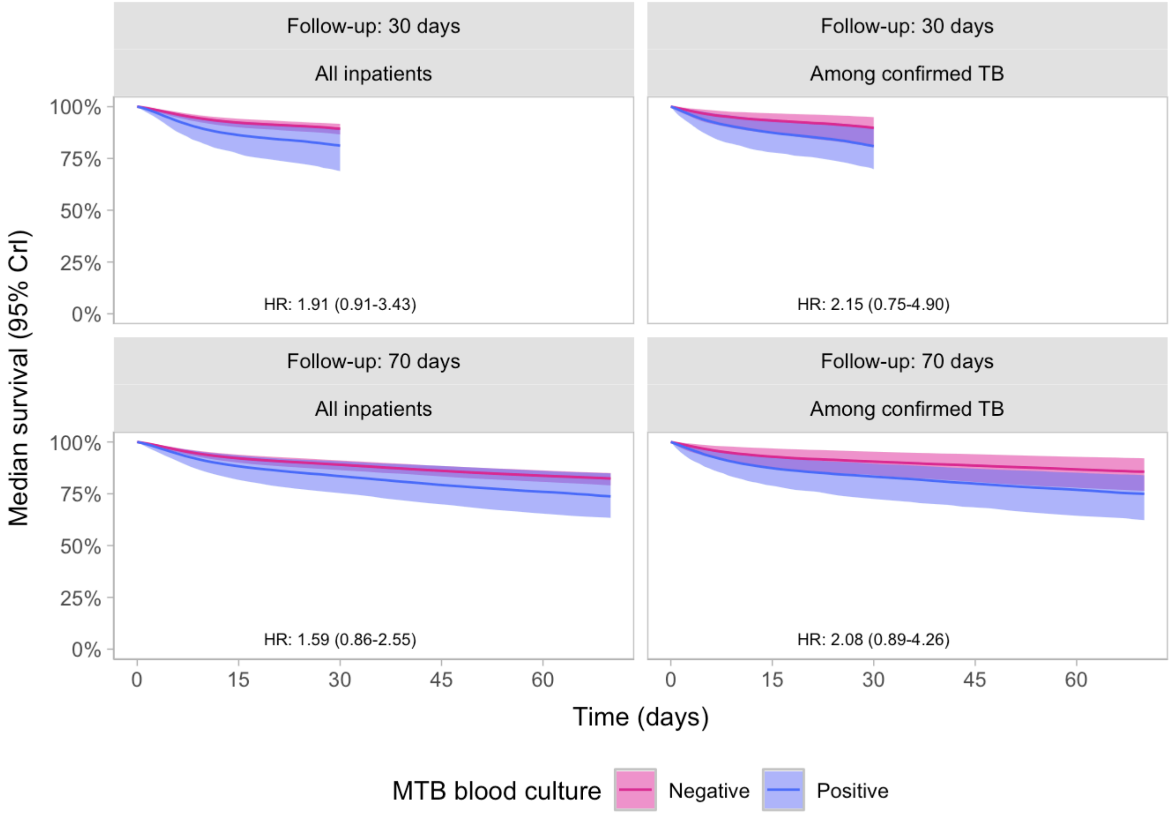

Supplementary Figure 3: Survival in sensitivity analysis with the expanded case definition of having either a mycobacterial blood culture positive for MTB, or having both urine Xpert Ultra and Determine-LAM tests positive

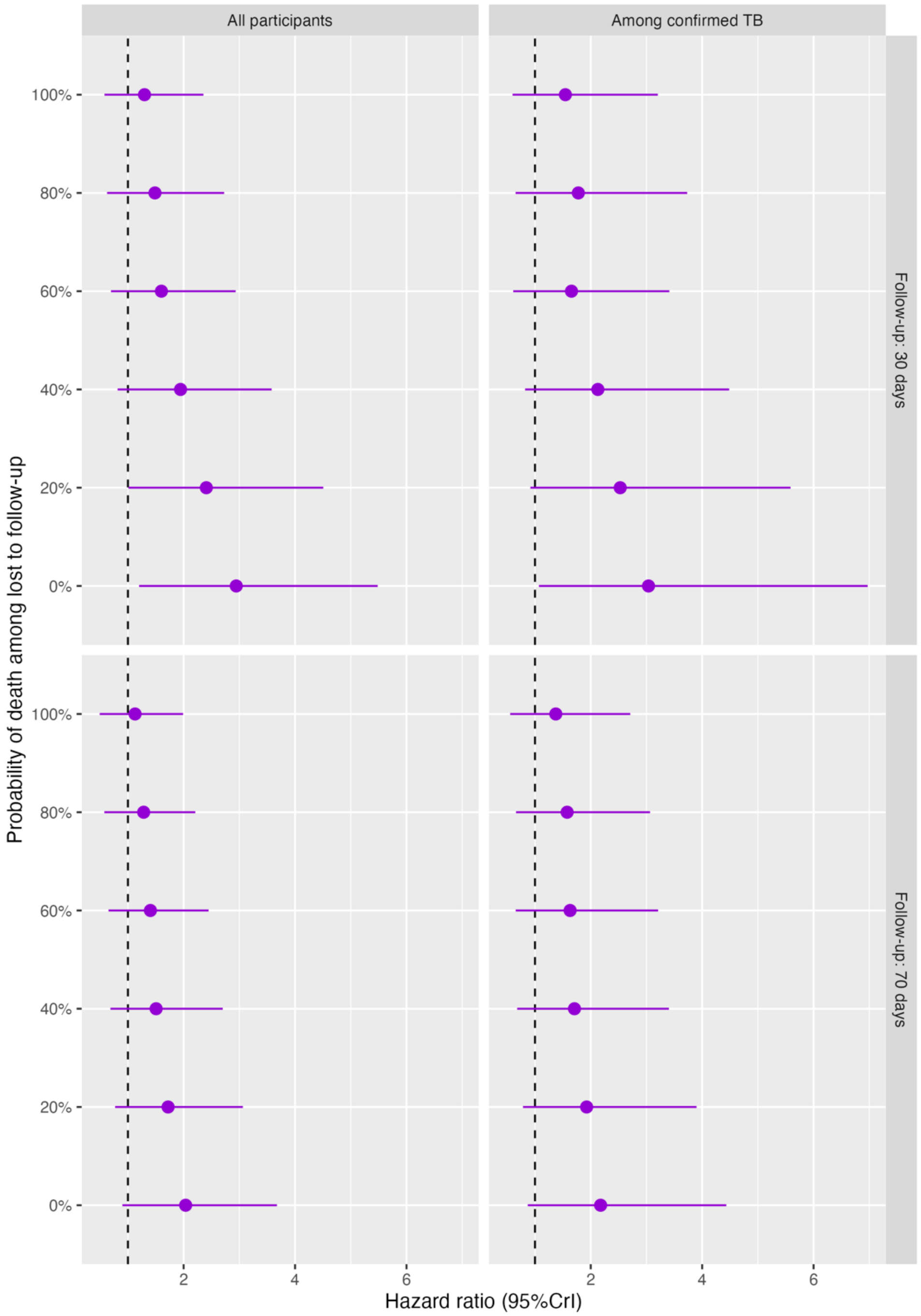

Supplementary figure 4: Sensitivity analysis of hazards of mortality according to the probability of death amongst those who were lost to follow up

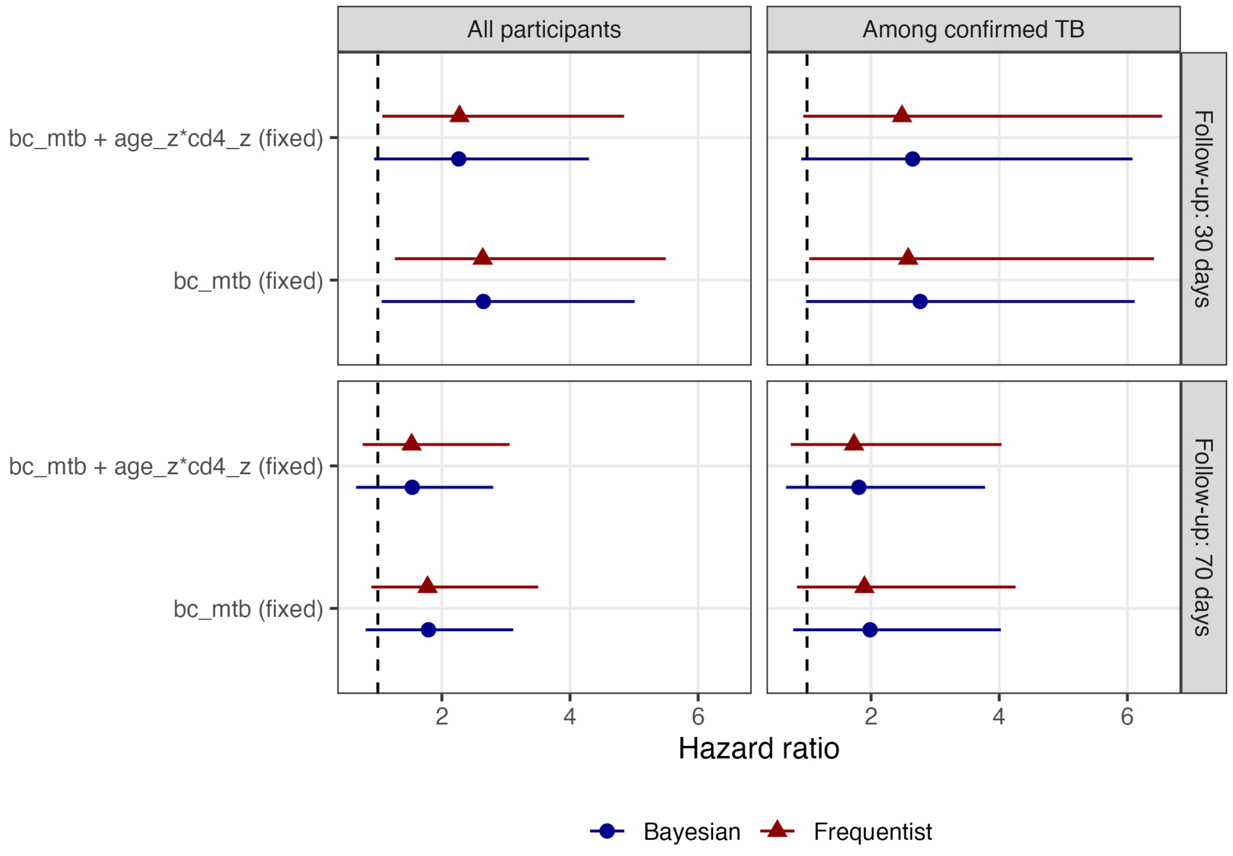

Supplementary Figure 5: Comparison of estimates from Bayesian and frequentist models in inpatients, with hazard ratios on the X axis and model structure on the Y axis

bc_mtb=MTB bloodstream infection, age_z = standardised age, cd4_z = standardised CD4 count
